# Supplementary material for: Single-molecule localization microscopy reveals STING clustering at the trans-Golgi network through palmitoylation-dependent accumulation of cholesterol
Source: Nat Commun. 2024 Jan 11;15:220. doi: 10.1038/s41467-023-44317-5 (PMC10784591; doi:10.1038/s41467-023-44317-5)
Supplement: Supplementary file 1 — Supplementary Information [file 41467_2023_44317_MOESM1_ESM.pdf]

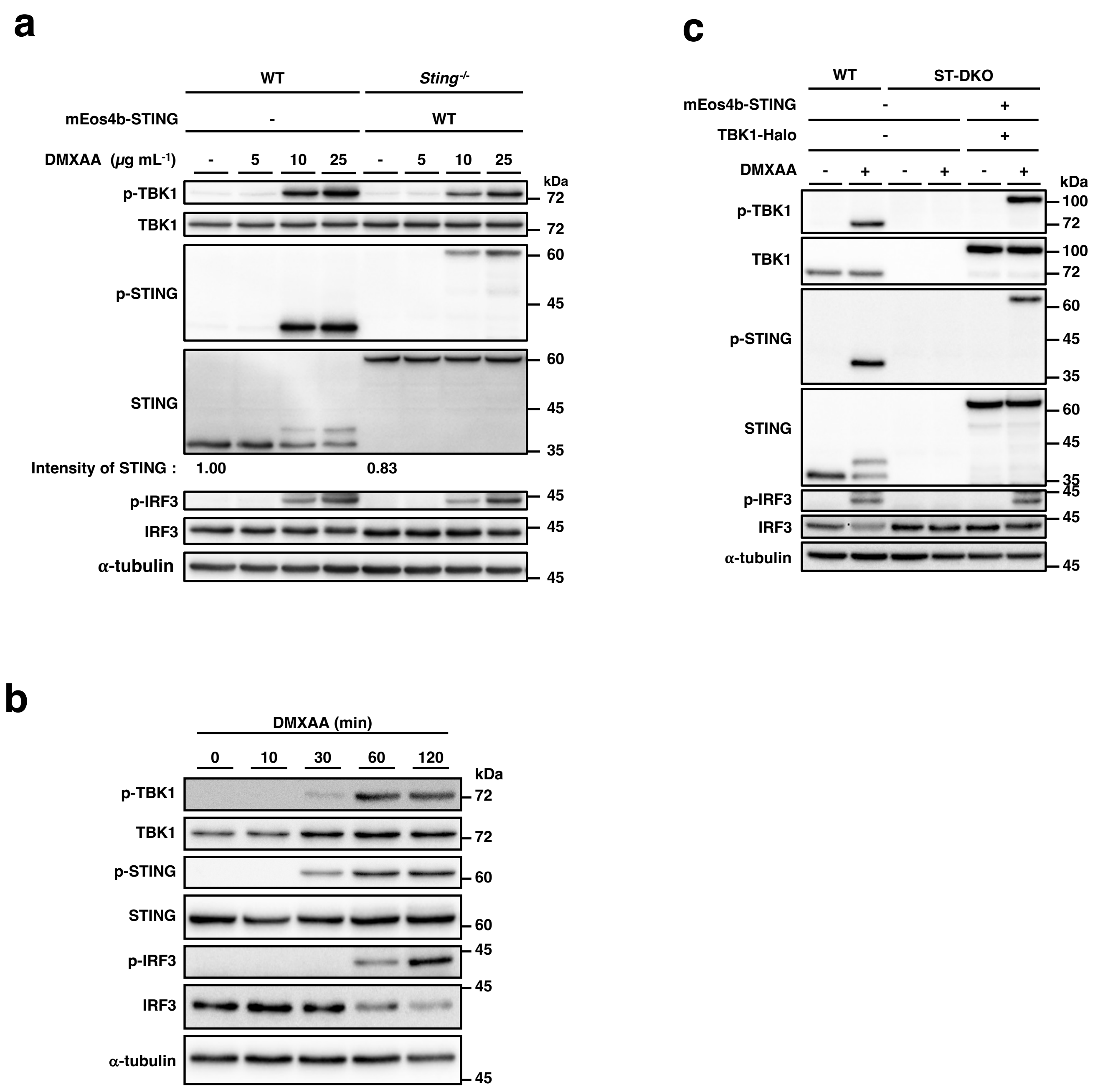

Supplementary Figure 1 | Validation of activity of mEos4b-STING and TBK1-Halo.
a, mEos4b-STING was stably expressed in *Sting*-/- MEFs. WT MEFs or mEos4b-STING-reconstituted *Sting*-/- MEFs were stimulated with DMXAA for 60 min. Cell lysates were then prepared and analyzed by western blot. **b**, mEos4b-STING-expressing *Sting*-/- MEFs were stimulated with DMXAA for the indicated time. Cell lysates were then prepared and analyzed by western blot. **c**, mEos4b-STING and TBK1-Halo were stably expressed in STING/TBK1-double knockout MEFs (ST-DKO MEFs). Cells were stimulated with DMXAA for 60 min. Cell lysates were then prepared and analyzed by western blot. Unprocessed blots are available in source data.

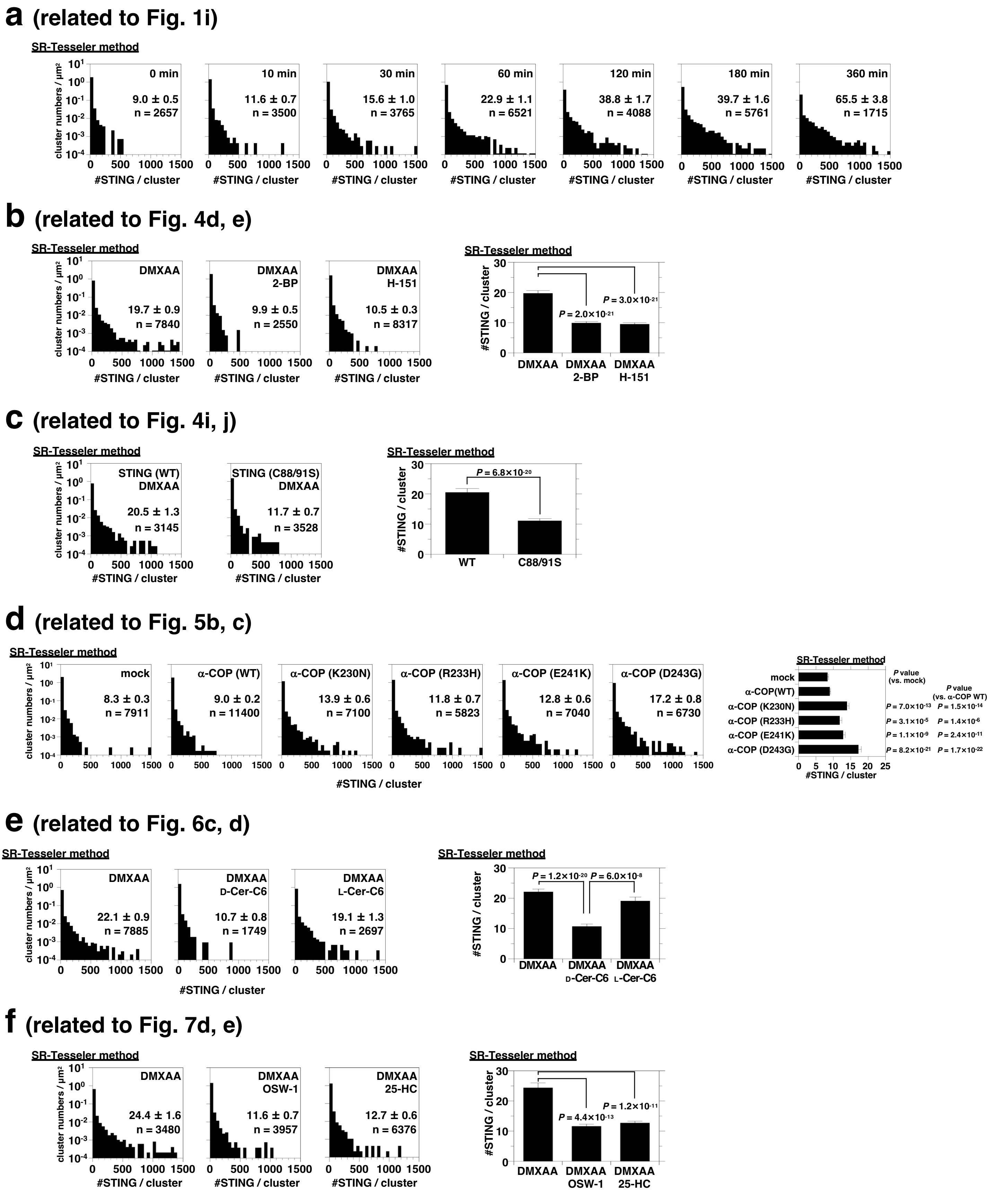

Supplementary Figure 2 | Cluster analysis of mEos4b-STING with SR-Tesseler method.
a, The distribution of [#STING/cluster] in cells stimulated with DMXAA for the indicated times. Data are presented as mean ± SEM. **b**, (left) The distribution of [#STING/cluster] and (right) the average of [#STING/cluster] in cells stimulated with DMXAA for 60 min in the presence of 2-BP or H-151. Data are presented as mean ± SEM. **c**, (left) The distribution of [#STING/cluster] and (right) the average of [#STING/cluster] of STING WT or C88/91S mutant in cells stimulated with DMXAA for 60 min. Data are presented as mean ± SEM. **d**, (left) The distribution of [#STING/cluster] and (right) the average of [#STING/cluster] in cells expressing α-COP variants. Data are presented as mean ± SEM. **e**, (left) The distribution of [#STING/cluster] and (right) the average of [#STING/cluster] in cells stimulated with DMXAA for 60 min in the presence of D-ceramide-C6 (D-Cer-C6) or L-ceramide-C6 (L-Cer-C6). Data are presented as mean ± SEM. **f**, (left) The distribution of [#STING/cluster] and (right) the average of [#STING/cluster] in cells stimulated with DMXAA for 60 min in the presence of OSW-1 or 25-HC. Data are presented as mean ± SEM. n indicates the number of examined clusters. The P-value (Welch's t-test, both-sided) was less than the significance level (**c**). The P-values (Welch's t-test, both-sided) were less than the significance levels corrected by the Holm-Sidak method (**b**, **d**, **e**, and **f**). Source numerical data are available in source data.

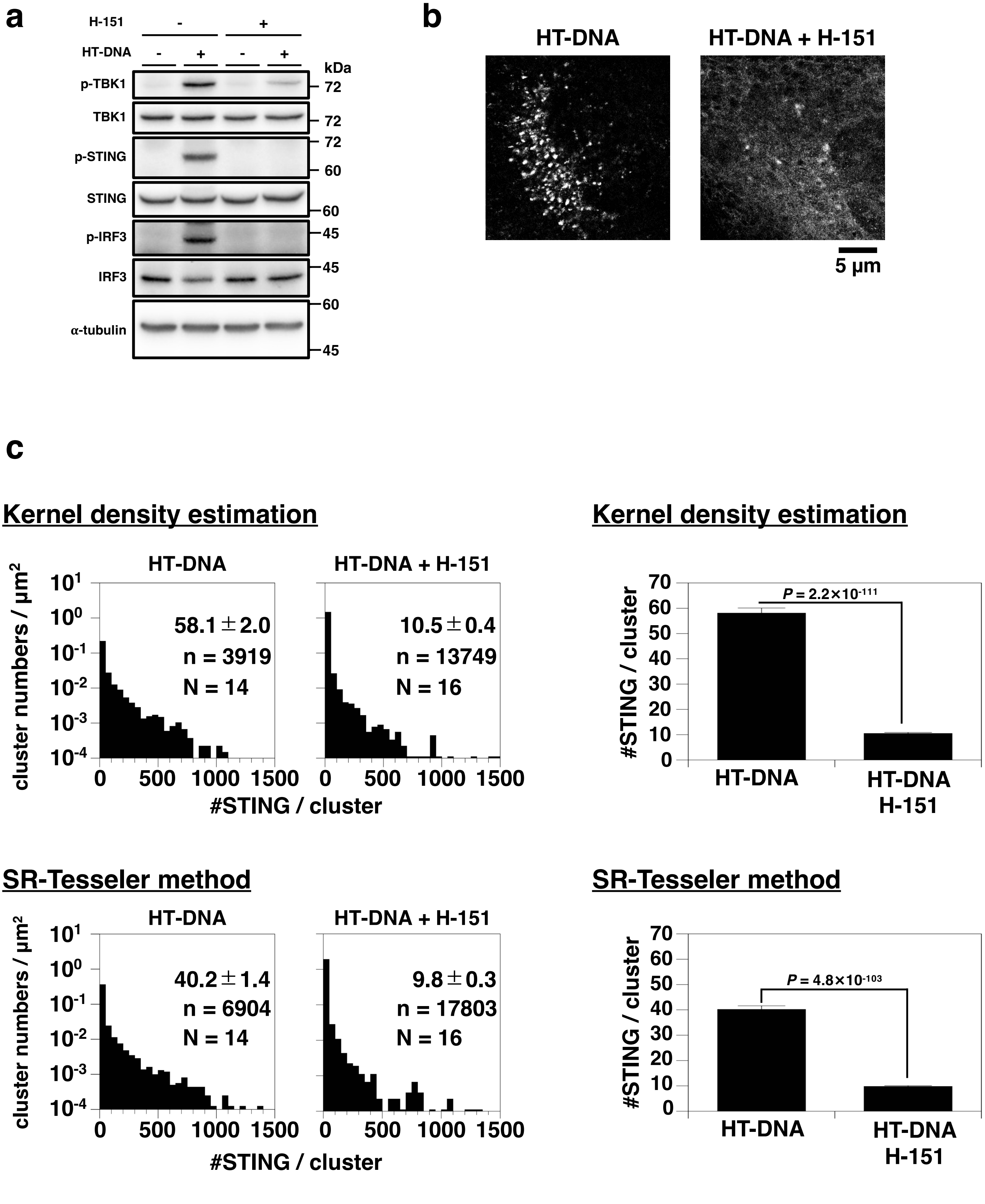

Supplementary Figure 3 | A natural ligand HT-DNA induced palmitoylation-dependent clustering of mEos4b-STING.
a, mEos4b-STING-expressing *Sting*$^{-/-}$ MEFs were pretreated with H-151 (10 μM) for 2 h and stimulated with HT-DNA (2 μg mL^{-1}) for 2 h. Cell lysates were prepared and analyzed by western blot. **b**, Typical PALM super-resolution images of mEos4b-STING. (left) mEos4b-STING-reconstituted *Sting*$^{-/-}$ MEFs were stimulated with HT-DNA (2 μg mL^{-1}) for 2 h. (right) The cells were treated with H-151 (10 μM) for 2 h before HT-DNA stimulation. **c**, (left) The distribution of [#STING/cluster] and (right) the average of [#STING/cluster] in cells stimulated with HT-DNA for 2 h with or without H-151. KDE (top) or SR-Tesseler (bottom) method was used for segmentation. n and N indicate the number of examined clusters and cells, respectively. Data are presented as mean ± SEM. The P-values (Welch's t-test, both-sided) were less than the significance level. Source numerical data and unprocessed blot are available in source data.

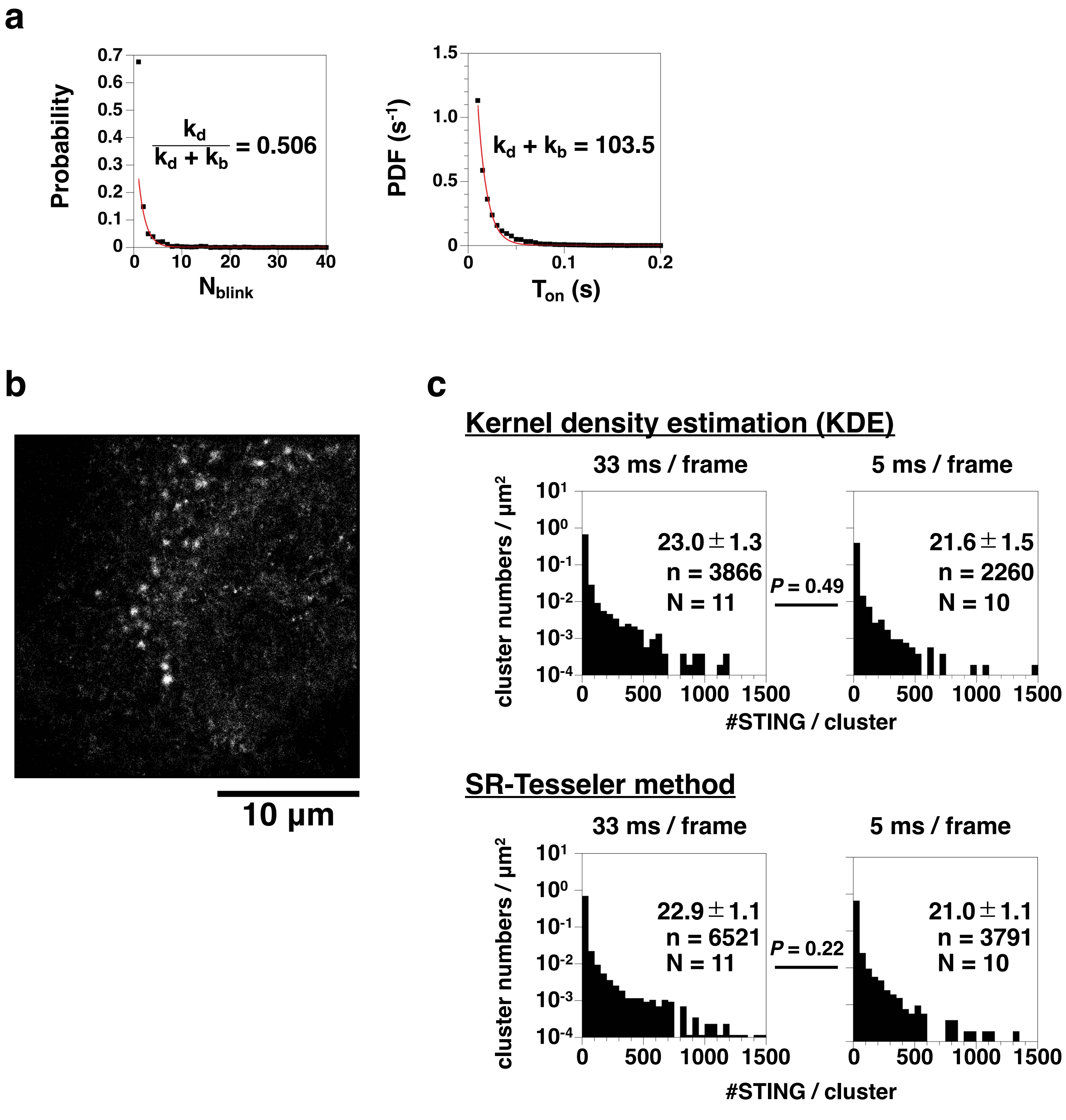

Supplementary Figure 4 | The number of mEos4b-STING molecules per cluster obtained by analyzing data acquired at 5 ms was not significantly different from those acquired at 33 ms.

a, (left) Distribution of N_{blink} fitted with a geometric distribution (red line). (right) T_{on} distribution of mEos4b follows a single exponential decay (red line). **b**, A typical PALM super-resolution image of mEosb-STING in cells stimulated with DMXAA (25 μg mL^{-1}) for 60 min at 37 °C. Data acquisition of live-cell PALM of mEos4b-STING for 5400 frames was performed at 5 ms resolution. **c**, The distribution of [#STING/cluster], which was obtained by analyzing data acquired at 33 ms (left) or 5 ms (right). KDE (top) or SR-Tesseler (bottom) method was used for segmentation. The number in the graph indicates mean ± SEM. n and N indicate the number of examined clusters and cells, respectively. P-values (Welch's t-test, both-sided) show no significant difference between the results obtained at 33 ms/frame and at 5 ms/frame. Source numerical data are available in source data.

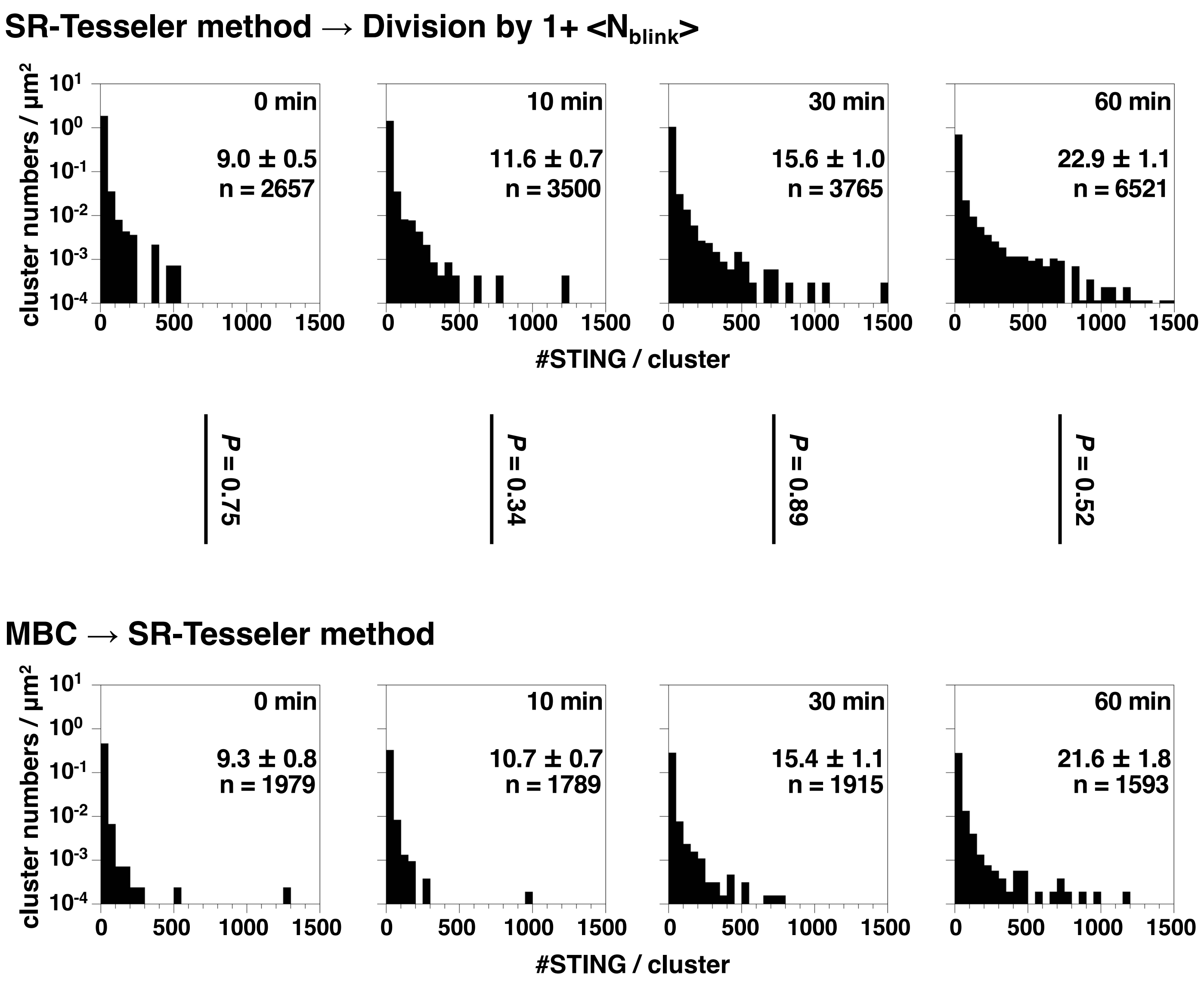

Supplementary Figure 5 | The distribution of [#STING/cluster] estimated by two methods of multiple-blinking correction.
mEos4b-STING-reconstituted STING-KO MEFs were stimulated with DMXAA (25 µg mL^{-1}) for 10, 30, and 60 min. The numbers of mEos4b-STING molecules per cluster were determined by dividing the numbers of localization segmented by the SR-Tesseler analysis with $1+<N_{blink}>$ (top), or by correcting multiple-blinking of mEos4b using model-based correction (MBC), followed by segmentation by the SR-Tesseler analysis (bottom). n indicates the number of examined clusters. The averaged numbers of mEos4b-STING molecules per cluster estimated by the two methods were not significantly different from each other in all the incubation periods ($p > 0.05$, Welch's T-test, both-sided). Data are presented as mean ± SEM. Source numerical data are available in source data.

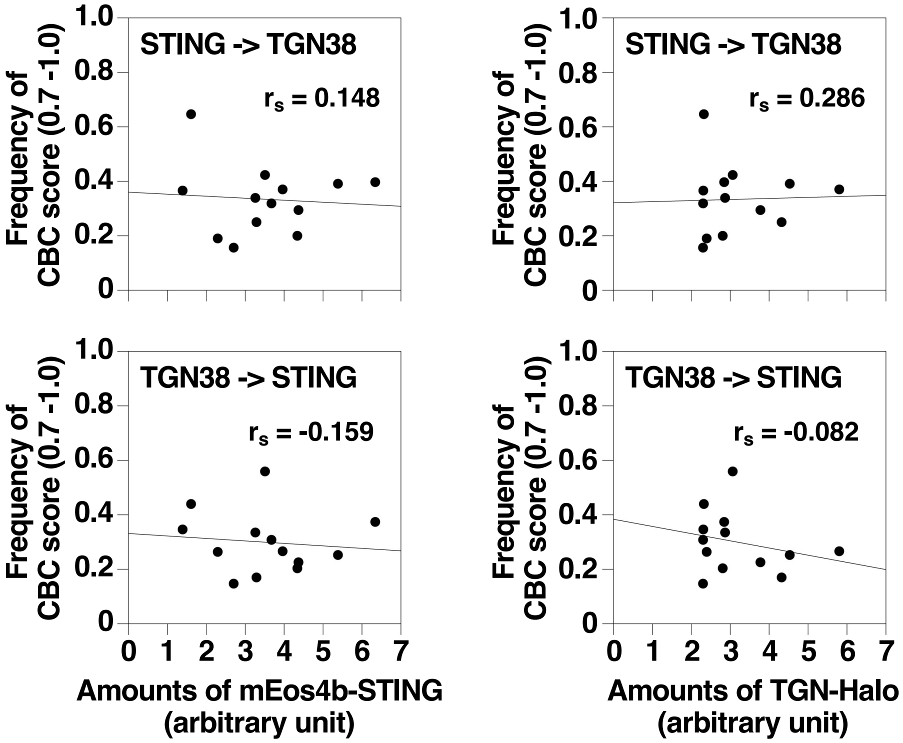

Supplementary Figure 6 | The expression levels of STING or TGN38 in cells observed in Fig. 2d exhibited only very weak correlation with the degree of colocalization between STING and TGN38.

mEos4b-STING- and TGN38-Halo-expressing *Sting*$^{-/-}$ MEFs were stimulated with DMXAA for 60 min. The summation of frequency in each bin of CBC scores between 0.7 and 1.0 was plotted for STING relative to TGN38 (top) or for TGN38 to STING (bottom) against amounts of mEos4b-STING (left) or TGN38-Halo (right) in cells observed in Fig. 2d. Spearman's rank correlation coefficients (r_s) for all the graphs fell within the range of -0.3 and 0.3, thereby indicating only very weak correlation. Source numerical data are available in source data.

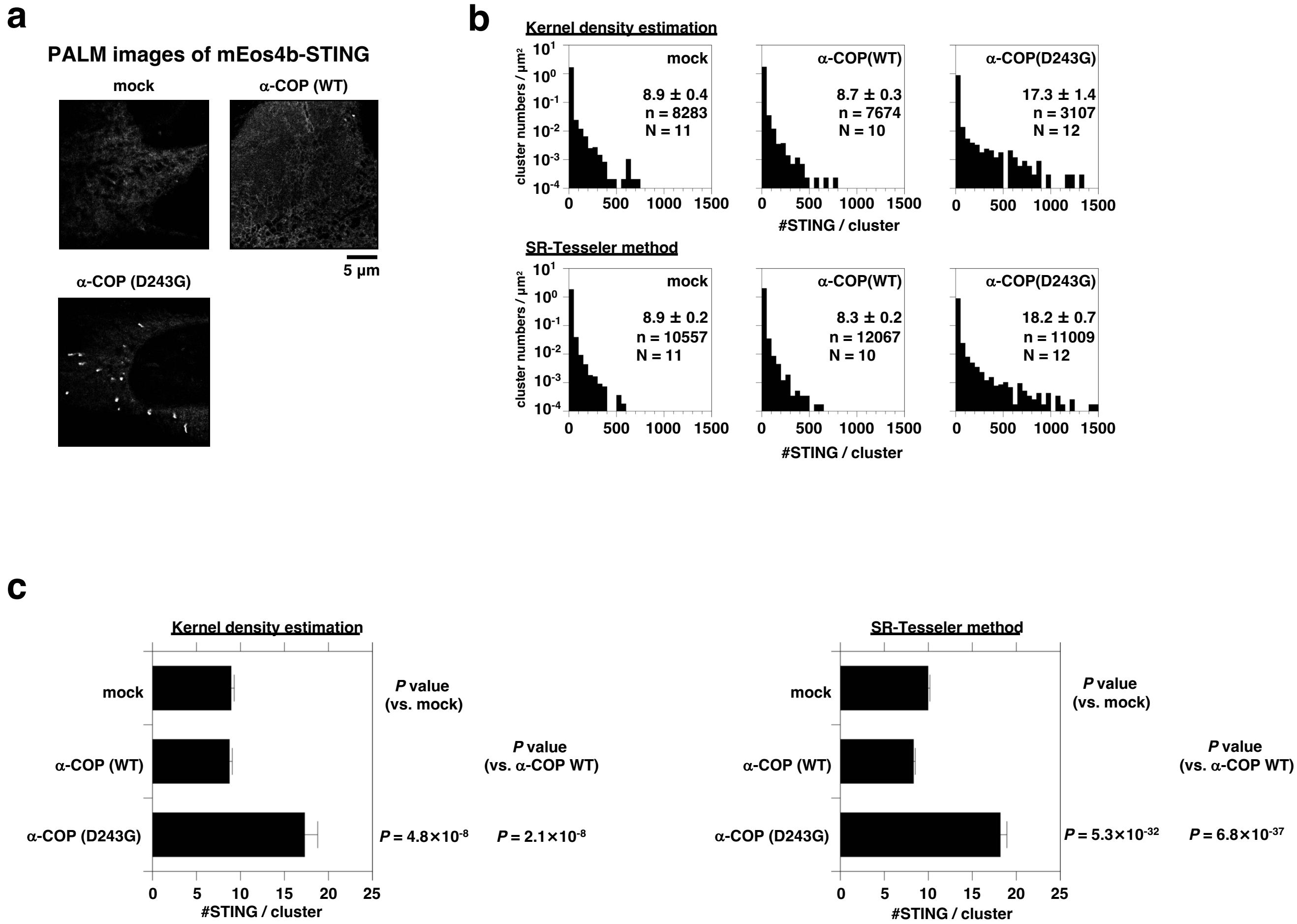

Supplementary Figure 7 | Expression of the α-COP variant in cGAS-KO cells induced the clustering of STING.
a, α-COP-FLAG and mEos4b-STING were stably expressed in cGAS-KO *Sting*$^{-/-}$ MEFs. Typical PALM images of mEos4b-STING in cells expressing wild-type α-COP or the disease-causative α-COP D243G variant. **b**, The distribution of [#STING/cluster] in (**a**). KDE (top) or SR-Tesseler (bottom) method was used for segmentation. n and N indicate the number of examined clusters and cells, respectively. **c**, The average of [#STING/cluster] in (**a**). Data are presented as mean ± SEM. The P-values (Welch's t-test, both-sided) were less than the significance level. Source numerical data are available in source data.

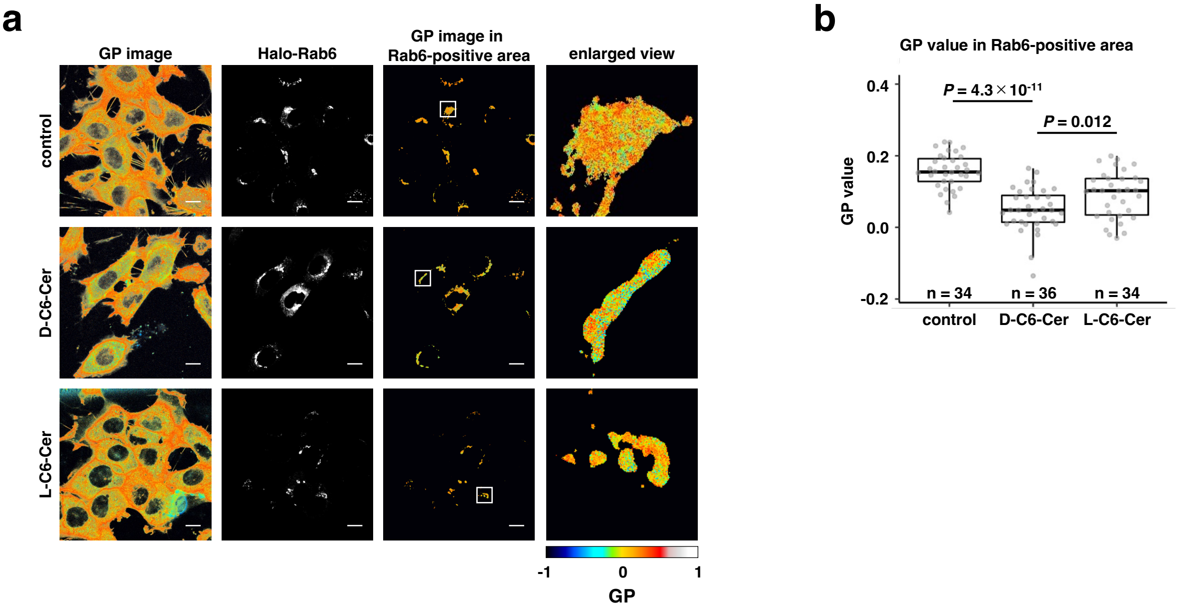

Supplementary Figure 8 | Supplementary data related to Figure 6.

a, Halo-Rab6-expressing MEFs were treated with D-Cer-C6 (20 µM) or L-Cer-C6 (20 µM) for 60 min, followed by treatment with HaloTag SaraFluor 650T Ligand (1 µM) and di-4-ANEPPDHQ (1 µg mL-1) for 30 min. Scale bars, 10 µm. **b**, Generalized polarization (GP) value of di-4-ANEPPDHQ in the Halo-Rab6-positive area of cells in (**a**) was measured. Data are presented in box-and-whisker plots with the minimum, maximum, sample median, first versus third quartiles and whiskers extend to a maximum of 1.5× interquartile range beyond the box. The data were statistically analyzed by performing one-way analysis of variance followed by Tukey-Kramer post hoc test for multiple comparisons. The sample size (n) represents the number of cells examined over 3 independent experiments. Source numerical data are available in source data.

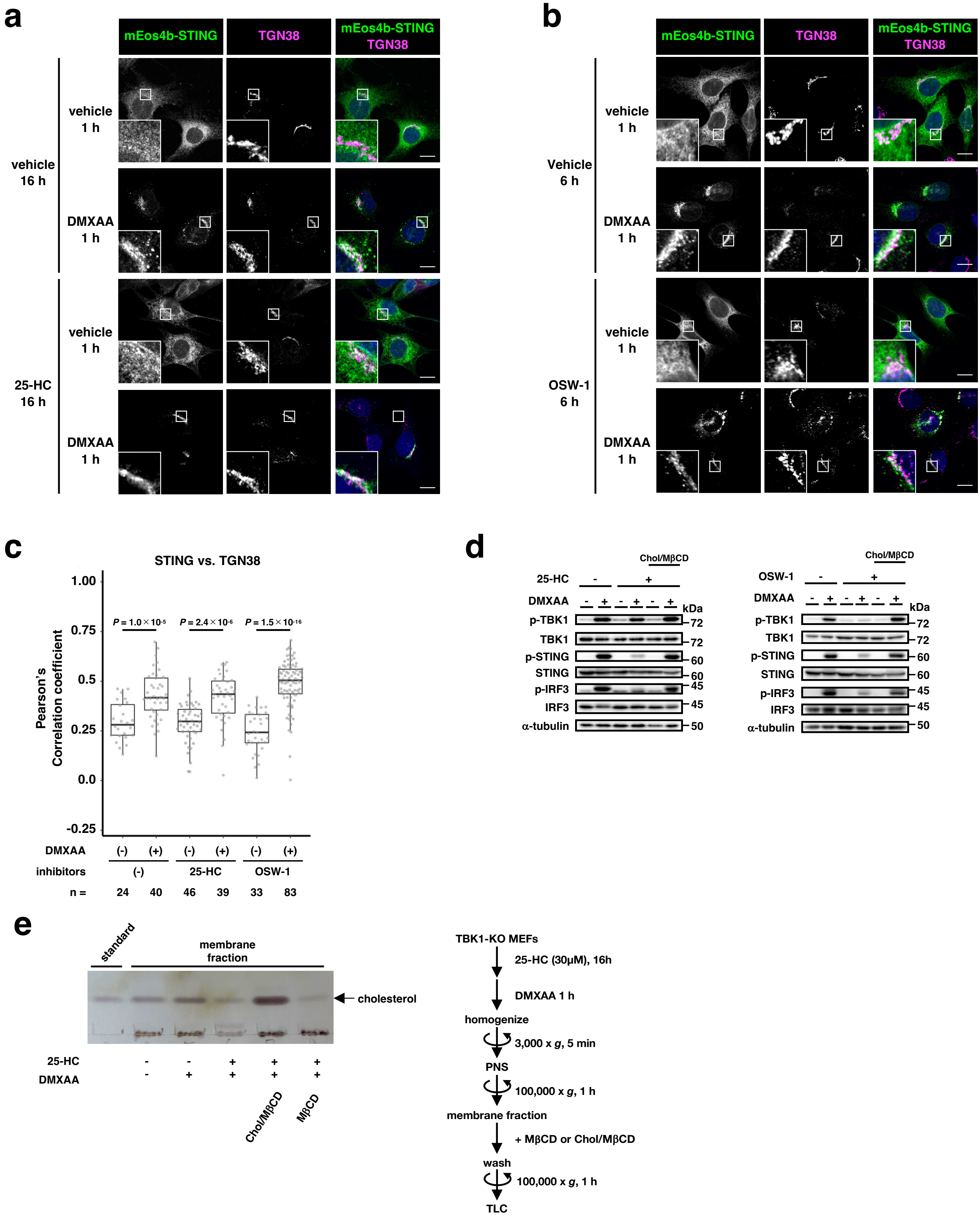

Supplementary Figure 9 | Supplementary data related to Figure 7.

a, **b**, mEos4b-STING-reconstituted *Sting*$^{-/-}$ MEFs were treated with (**a**) 25-HC (30 μM) for 16 h or (**b**) OSW-1 (0.125 nM) for 6 h, followed by stimulation with DMXAA (25 μg mL^{-1}) for 60 min. Cells were fixed, permeabilized, and stained for TGN38. DAPI (blue). Scale bars, 10 μm. **c**, The Pearson's correlation coefficient between mEos4b-STING and TGN38 in (**a** and **b**). Data are presented in box-and-whisker plots with the minimum, maximum, sample median, first versus third quartiles and whiskers extend to a maximum of 1.5× interquartile range beyond the box. The data were statistically analyzed by performing one-way analysis of variance followed by Welch's unpaired two-tailed *t*-test. The sample size (n) represents the number of cells examined over 3 independent experiments. **d**, mEos4b-STING-reconstituted *Sting*$^{-/-}$ MEFs were treated with (left) 25-HC (30 μM) for 16 h or (right) OSW-1 (0.125 nM) for 6 h, and then incubated with medium containing cholesterol-methyl-β-cyclodextrin complex (Chol/MβCD) (3.9 mM) for 3h, followed by stimulation with DMXAA (25 μg mL^{-1}) for 60 min. Cell lysates were then prepared and analyzed by western blot. **e**, Thin-layer chromatogram (TLC) analysis of total sterols in membrane fraction with TBK1 KO MEFs. Cholesterol contents were analyzed by TLC as described in Methods. Source numerical data and unprocessed blot are available in source data.

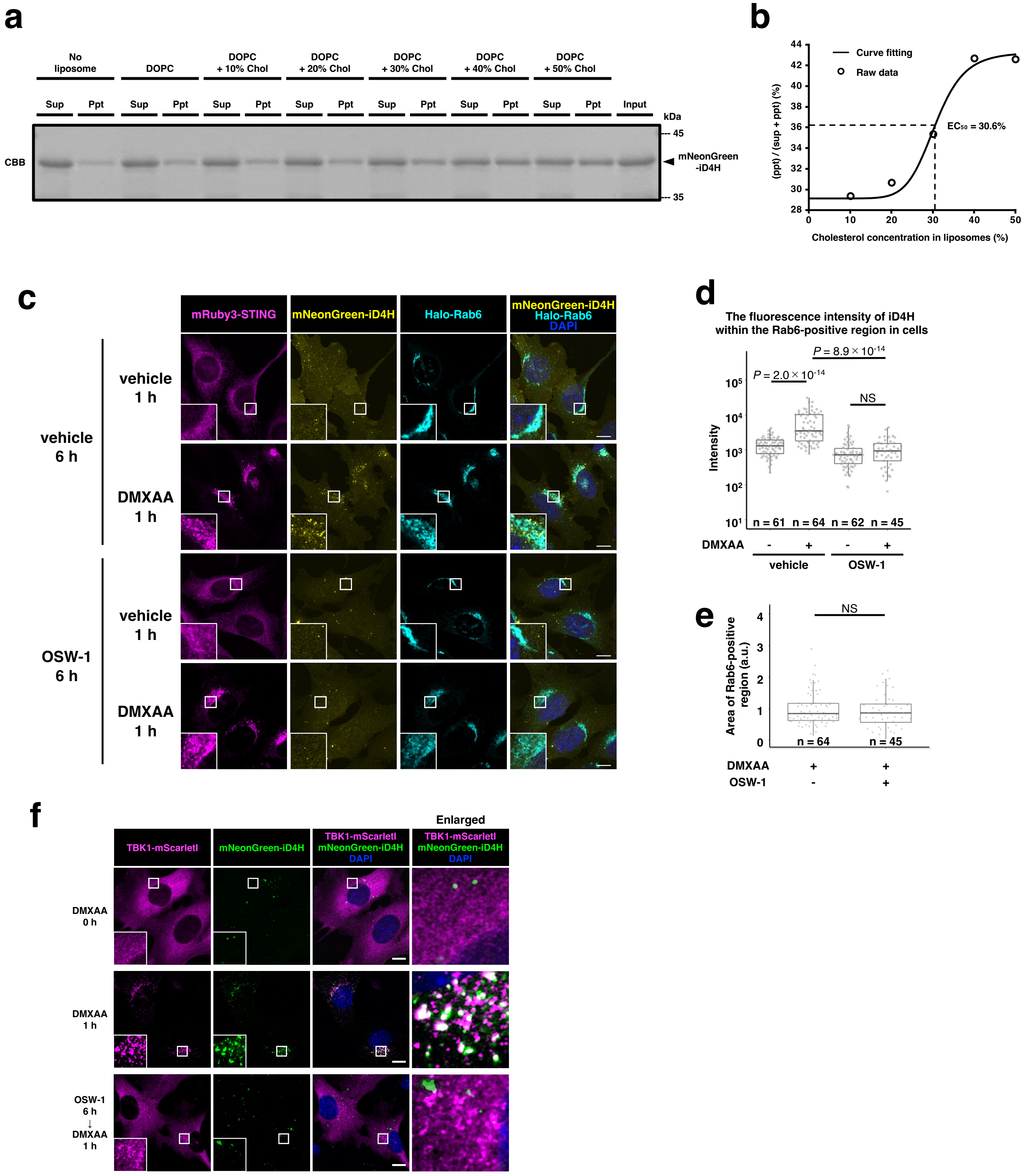

Supplementary Figure 10 | Supplementary data related to Figure 7.
a, mNeonGreen-iD4H recombinant protein was mixed with phosphatidylcholine-based liposomes containing the indicated % (mol/mol) of cholesterol. After 30 min, the mixture was spun at 20,000 x g for 30 min, and the resultant supernatant (Sup) and pellet (Ppt) were subjected to SDS-PAGE and Coomassie Brilliant Blue (CBB) Staining. **b**, The intensities of individual bands in (**a**) were quantified with Fiji, and the percentage of bound proteins was calculated. **c**, *Sting*$^{-/-}$ MEFs expressing mRuby3-STING (magenta), mNeonGreen-iD4H (yellow) and Halo-Rab6 (cyan) were pretreated with OSW-1 (0.125 nM) for 6 h, and then stimulated with DMXAA for 1 h. Cells were fixed, permeabilized, and stained with HaloTag SaraFluor 650T Ligand and with DAPI (blue). **d**, **e**, The fluorescence intensity of mNeonGreen-iD4H within the Rab6-positive region (**d**) or area of Rab6-positive region (**e**) in cells observed in (**c**) was quantified. Data are presented in box-and-whisker plots with the minimum, maximum, sample median, first versus third quartiles and whiskers extend to a maximum of 1.5× interquartile range beyond the box. The data were statistically analyzed by performing one-way analysis of variance followed by Tukey-Kramer post hoc test for multiple comparisons (**d**) or Welch's unpaired two-tailed *t*-test (**e**). The sample size (n) represents the number of cells examined over 3 independent experiments. NS, Not significant. a.u., Arbitrary Unit. **f**, TBK1 knock-out MEFs expressing TBK1-mScarletI (magenta) and mNeonGreen-iD4H (green) were pretreated with OSW-1 (0.125 nM) for 6 h, and then stimulated with DMXAA for 1 h. DAPI (blue). Scale bars, 10 µm (**c**, **f**). Source numerical data and unprocessed blot are available in source data.

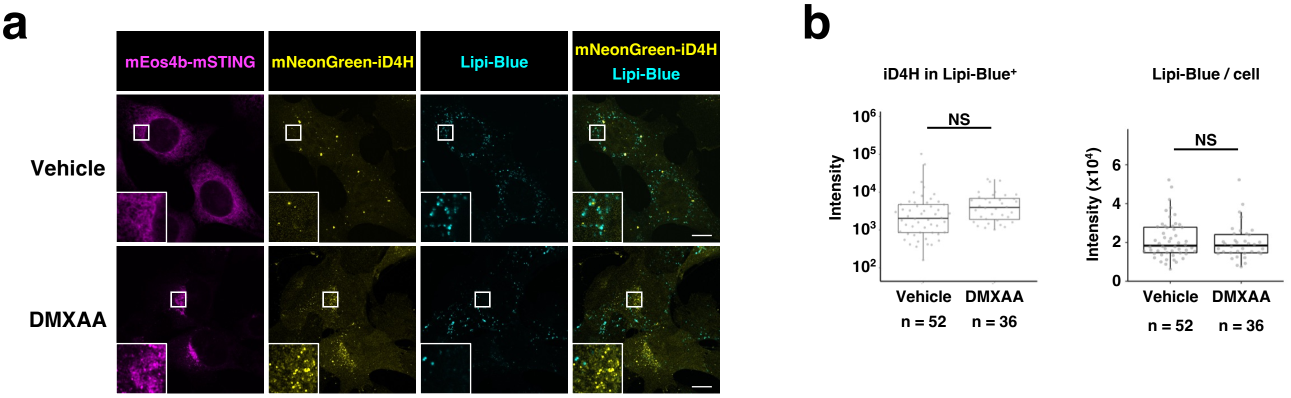

Supplementary Figure 11| Supplementary data related to Figure 7.
a, *Sting*$^{-/-}$ MEFs expressing mRuby3-STING (magenta) and mNeonGreen-iD4H (yellow) were stimulated with DMXAA for 60 min. Cells were fixed and stained with Lipi-Blue. Scale bars, 10 µm. **b**, (left) The fluorescence intensity of mNeonGreen-iD4H in Lipi-Blue-positive area (Lipi Blue^{+}) or (right) the fluorescence intensity of Lipi-Blue per cell in (**a**) was quantified. Data are presented in box-and-whisker plots with the minimum, maximum, sample median, first versus third quartiles and whiskers extend to a maximum of 1.5× interquartile range beyond the box. The data were statistically analyzed by performing Welch's unpaired two-tailed *t*-test. The sample size (n) represents the number of cells examined over 3 independent experiments. NS, not significant. Source numerical data are available in source data.

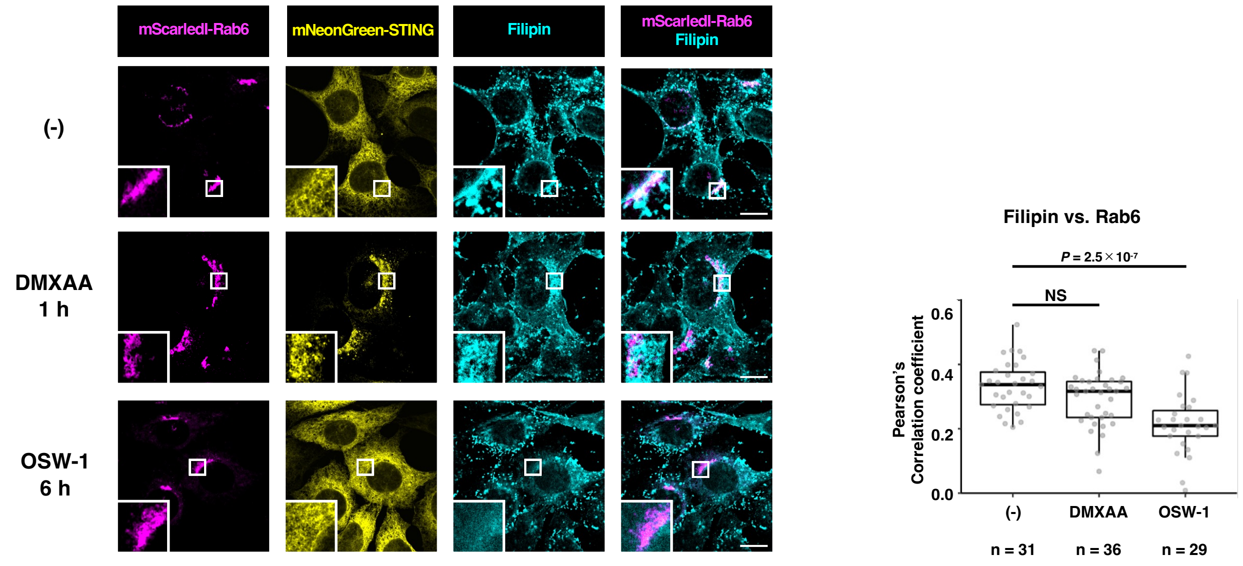

Supplementary Figure 12 | Supplementary data related to Figure 7.
(left) mNeongreen-STING- and mScarletI-Rab6-reconstituted *Sting*$^{-/-}$ MEFs were treated with DMXAA (25 µg mL^{-1}) or OSW-1 (0.125 nM) for the indicated times. Cells were fixed, permeabilized, and stained with filipin. Scale bars, 10 µm. (right) The Pearson's correlation coefficient between mNeongreen-STING and mScarletI-Rab6 in the left images. Data are presented in box-and-whisker plots with the minimum, maximum, sample median, first versus third quartiles and whiskers extend to a maximum of 1.5× interquartile range beyond the box. The data were statistically analyzed by performing one-way analysis of variance followed by Tukey-Kramer post hoc test for multiple comparisons. The sample size (n) represents the number of cells examined over 3 independent experiments. NS, not significant. Source numerical data are available in source data.

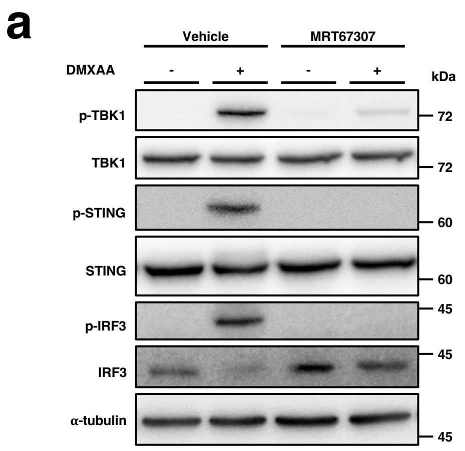

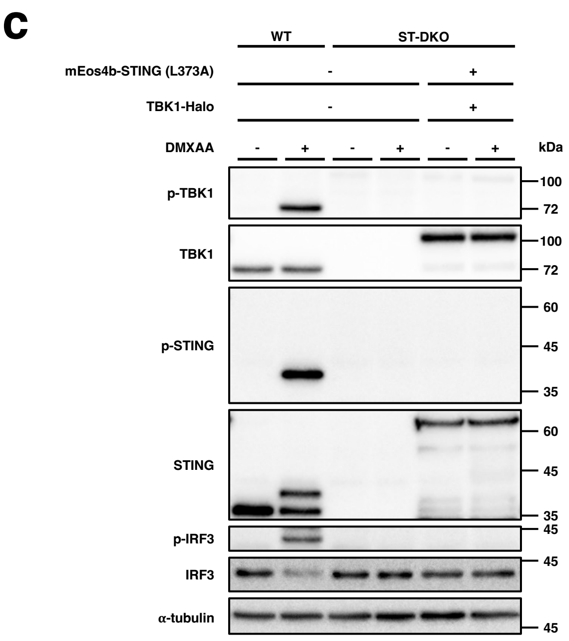

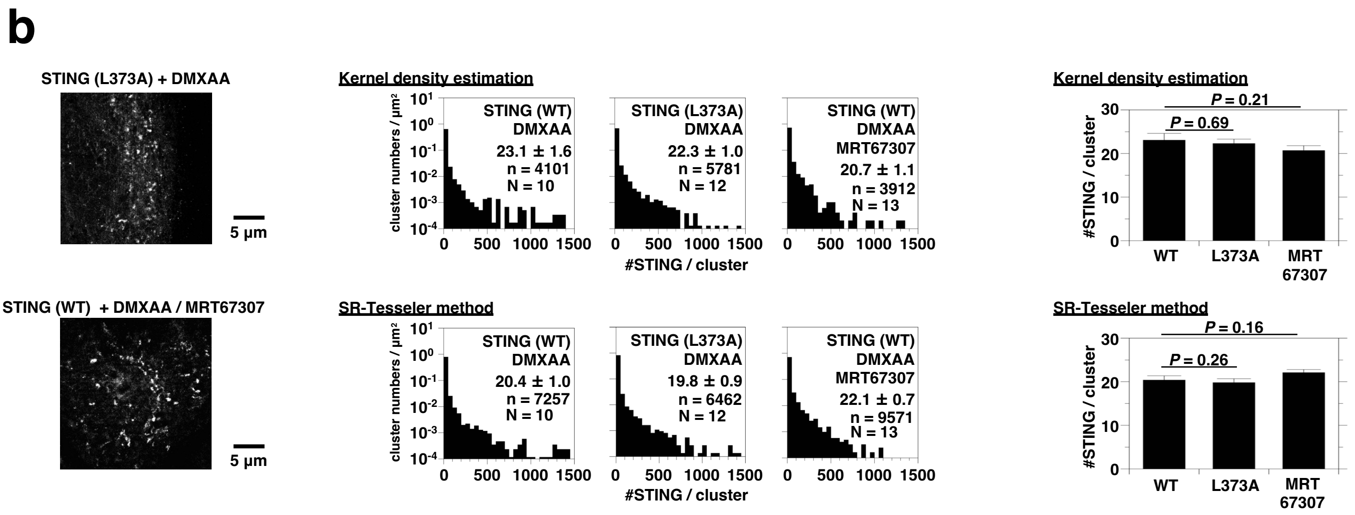

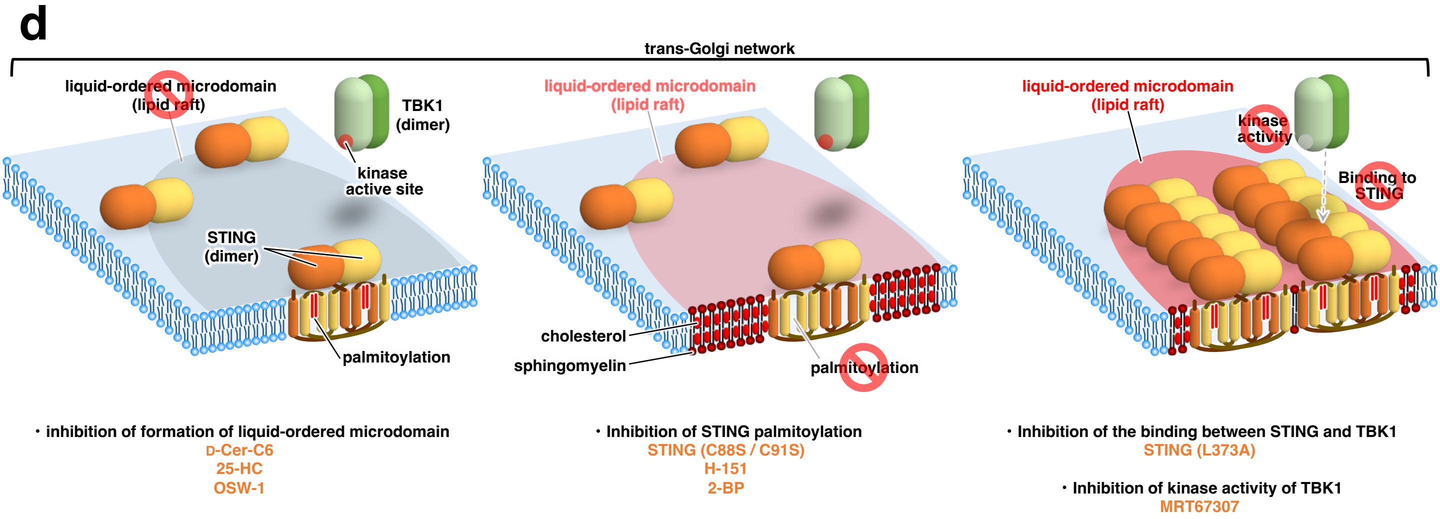

Supplementary Figure 13 | Supplementary data related to Figure 8.
a, mEos4b-STING-expressing *Sting*$^{-/-}$ MEFs were treated with MRT67307 (10 μM) for 2 h and stimulated with DMXAA for 60 min. Cell lysates were prepared and analyzed by western blot. **b**, (left) Typical PALM image of L373A mutant in cells stimulated with DMXAA for 60 min and that of wild-type STING in cells treated with DMXAA for 60 min in the presence of MRT67307. (middle) The distribution of [#STING/cluster]. n and N indicate the number of examined clusters and cells, respectively. (right) The average of [#STING/cluster]. The data are presented as mean ± SEM. Welch's t-test (both-sided) corrected by the Holm-Sidak method revealed no significant difference. **c**, mEos4b-STING (L373A) and TBK1-Halo were stably expressed in STING/TBK1-double knockout MEFs. Cells were stimulated with DMXAA for 60 min. Cell lysates were then prepared and analyzed by western blot. **d**, A graphical abstract illustrating cholesterol- and palmitoylation-dependent STING clustering at the trans-Golgi network. Source numerical data and unprocessed blot are available in source data.

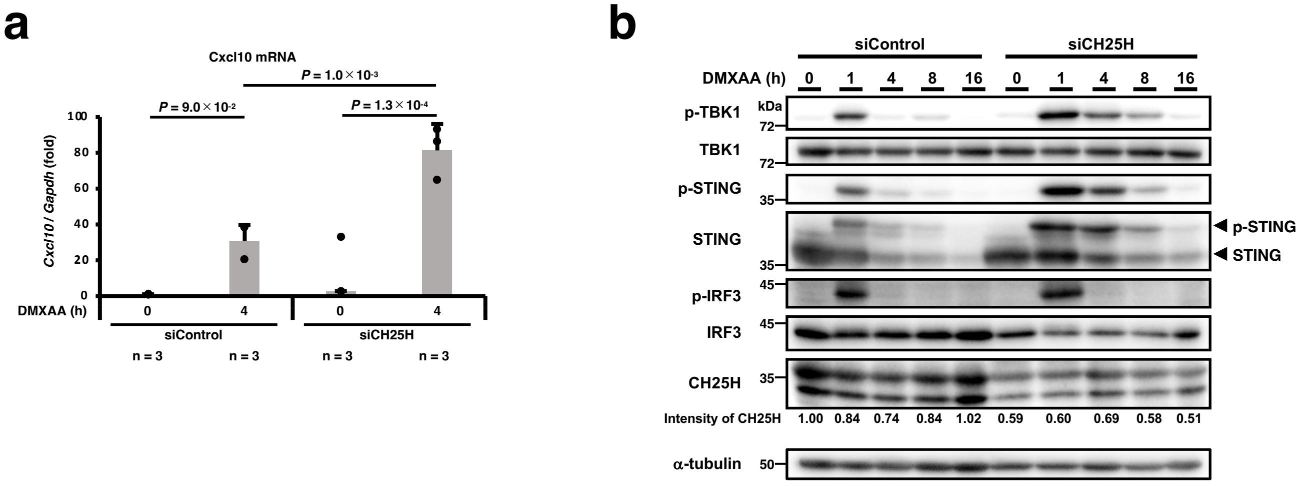

Supplementary Figure 14 | Knockdown of CH25H enhanced the STING signaling.

a, STING (WT) was stably expressed in B16-Blue ISG-KO-STING cells. Cells were treated with the indicated siRNAs for 72 h followed by stimulation with DMXAA (25 μg mL^{-1}) for 4 h. The expression of Cxcl10 was quantified by qRT-PCR. Data are presented as mean values ± standard deviation. The data were statistically analyzed by performing one-way analysis of variance followed by Tukey-Kramer post hoc test for multiple comparisons. The sample size (n) represents the biological replicates. **b**, Cells in (**a**) were treated with the indicated siRNAs for 72 h, followed by DMXAA (25 μg mL^{-1}) stimulation for the indicated times. Cell lysates were prepared and analyzed by western blot. Source numerical data and unprocessed blots are available in source data.
