# Supplementary material for: Single-molecule localization microscopy reveals STING clustering at the trans-Golgi network through palmitoylation-dependent accumulation of cholesterol
Source: Nat Commun. 2024 Jan 11;15:220. doi: 10.1038/s41467-023-44317-5 (PMC10784591; doi:10.1038/s41467-023-44317-5)
Supplement: Supplementary file 3 — Description of Additional Supplementary Files [file 41467_2023_44317_MOESM3_ESM.pdf]

Description of Additional Supplementary Files

Supplementary Movie 1

TBK1-Halo-reconstituted TBK1-KO MEFs were treated with di-4-ANEPPDHQ (1 µg mL^{-1}) and HaloTag SaraFluor 650T Ligand (1 µM) for 30 min, followed by stimulation with DMXAA (25 µg ml^{-1}) for 120 min. Live-cell images were taken at 5-min intervals by confocal microscopy. The mean GP value in the cytoplasm (Cyto) or in TBK1 foci was quantified. Data are presented in box-and-whisker plots with the minimum, maximum, sample median, first versus third quartiles and whiskers extend to a maximum of 1.5× interquartile range beyond the box. The corresponding data points are overlaid on the plots. The data were statistically analyzed by performing one-way analysis of variance followed by Tukey-Kramer post hoc test for multiple comparisons. The sample size (n) represents the number of cells examined over 3 independent experiments. Source numerical data are available in source data.

Supplementary Movie 2

A representative simultaneous two-colour imaging video clip of mEos4b-STING clusters (green, PALM image) and single-molecules of TBK1-Halo-SF650T (magenta) in a living cell. Arrowheads indicate colocalization. The video was recorded at video rate and replayed in real-time. Scale bar 2 µm.
