# Supplementary material for: Single-molecule localization microscopy reveals STING clustering at the trans-Golgi network through palmitoylation-dependent accumulation of cholesterol
Source: Nat Commun. 2024 Jan 11;15:220. doi: 10.1038/s41467-023-44317-5 (PMC10784591; doi:10.1038/s41467-023-44317-5)
Supplement: Supplementary file 6 — Reporting Summary [file 41467_2023_44317_MOESM6_ESM.pdf]

nature portfolio

Corresponding author(s): Tomohiko Taguchi

Last updated by author(s): 12/07/2023

Reporting Summary

Nature Portfolio wishes to improve the reproducibility of the work that we publish. This form provides structure for consistency and transparency in reporting. For further information on Nature Portfolio policies, see our Editorial Policies and the Editorial Policy Checklist.

Statistics

For all statistical analyses, confirm that the following items are present in the figure legend, table legend, main text, or Methods section.

n/a	Confirmed	
☐	☒	The exact sample size (*n*) for each experimental group/condition, given as a discrete number and unit of measurement
☐	☒	A statement on whether measurements were taken from distinct samples or whether the same sample was measured repeatedly
☐	☒	The statistical test(s) used AND whether they are one- or two-sided<br>*Only common tests should be described solely by name; describe more complex techniques in the Methods section.*
☐	☒	A description of all covariates tested
☐	☒	A description of any assumptions or corrections, such as tests of normality and adjustment for multiple comparisons
☐	☒	A full description of the statistical parameters including central tendency (e.g. means) or other basic estimates (e.g. regression coefficient) AND variation (e.g. standard deviation) or associated estimates of uncertainty (e.g. confidence intervals)
☐	☒	For null hypothesis testing, the test statistic (e.g. *F*, *t*, *r*) with confidence intervals, effect sizes, degrees of freedom and *P* value noted<br>*Give P values as exact values whenever suitable.*
☒	☐	For Bayesian analysis, information on the choice of priors and Markov chain Monte Carlo settings
☒	☐	For hierarchical and complex designs, identification of the appropriate level for tests and full reporting of outcomes
☐	☒	Estimates of effect sizes (e.g. Cohen's *d*, Pearson's *r*), indicating how they were calculated

Our web collection on statistics for biologists contains articles on many of the points above.

Software and code

Policy information about availability of computer code

Data collection	Western blot data were collected using FUSION SOLO (software;Evolution Capt). Microscopy data were collected using Zeiss ZEN 2.3 SP1 FP3 (black, 64 bit) (ver. 14.0.21.201). Quantitative real-time PCR (qRT-PCR) was performed using LightCycler 96 (Roche). TIRF microscopy:Home-built single-molecule imaging station built on Olympus IX-83 (Olympus) (Konishi et al., J. Org. Chem., 2020; Takahashi et al., RSC Chem. Biol. 2022). Single fluorescent-molecule tracking: WinTrack, WinATR, and WinSAT, produced in house (Komura et al., Nat. Chem. Biol. 2016; Kinoshita et al., J. Cell Biol. 2017; Morise et al., Nat. Commun. 2019). Acquisitions of super-resolution microscopic images: ThunderSTORM plugin of Fiji (ver. 2.1.0/1.54f) (Fujiwara et al., J. Cell Biol. 2023a, b)
Data analysis	Data were analysed by Fiji (ver. 2.1.0/1.53c) for the intensity of the bands of western blot, by Fiji (ver. 2.1.0/1.53c) including the Trainable Weka Segmentation plugin (v3.3.4), Cellpose (v1.0), R (ver. 4.1.2), and KNIME (ver. 4.5.1) for co-localization analysis of confocal images, by the GDSC SMLM plugin of Fiji (ver. 2.1.0/1.53c) for the estimation of the parameters from the obtained images of mEos4b immobilized in PVA, or by the ThunderSTORM plugin (ver.1.3) of Fiji (ver. 2.1.0/1.53c) for the detection of the fluorescent spots in the images. No custom-made code was created to analyze super-resolution microscopic images. Statistical analysis was performed by Origin Pro 2018b (OriginLab), Statview 5.0. (SAS).

For manuscripts utilizing custom algorithms or software that are central to the research but not yet described in published literature, software must be made available to editors and reviewers. We strongly encourage code deposition in a community repository (e.g. GitHub). See the Nature Portfolio guidelines for submitting code & software for further information.

Data

Policy information about availability of data

All manuscripts must include a data availability statement. This statement should provide the following information, where applicable:

- Accession codes, unique identifiers, or web links for publicly available datasets
- A description of any restrictions on data availability
- For clinical datasets or third party data, please ensure that the statement adheres to our policy

The data sets generated during and/or analyzed during the current study are available from the corresponding authors upon reasonable request. The information of following genes was obtained from NCBI nr database (https://www.ncbi.nlm.nih.gov): mouse STING (NM_028261), mouse TBK1 (NM_019786), mouse GM130 (NM_001080968.2), mouse TfnR (Transferrin Receptor) (NM_011638.4), mouse TGN38 (NM_009443.3), human TBK1 (NM_013254), mouse Rab6a (NM_024287), mouse Rab11a (NM_017382.5), mouse α-COP (NM_009938.4), and mouse cavin1 (NM_008986.2).

Research involving human participants, their data, or biological material

Policy information about studies with human participants or human data. See also policy information about sex, gender (identity/presentation), and sexual orientation and race, ethnicity and racism.

Reporting on sex and gender	N.A.
Reporting on race, ethnicity, or other socially relevant groupings	N.A.
Population characteristics	N.A.
Recruitment	N.A.
Ethics oversight	N.A.

Note that full information on the approval of the study protocol must also be provided in the manuscript.

Field-specific reporting

Please select the one below that is the best fit for your research. If you are not sure, read the appropriate sections before making your selection.

[x] Life sciences   [ ] Behavioural & social sciences   [ ] Ecological, evolutionary & environmental sciences

For a reference copy of the document with all sections, see nature.com/documents/nr-reporting-summary-flat.pdf

Life sciences study design

All studies must disclose on these points even when the disclosure is negative.

Sample size	No sample size calculation was applied in this study to predetermine sample sizes for experiments using cell lines. A sample size of three or more was used as to evaluate the spread of the data and was determined based upon other studies with similar methodologies (PMID: 27324217, 29093443, 33397928)
Data exclusions	No data have been excluded from any analysis.
Replication	All experiments have been repeated at least three times independently, and each yielding similar results.
Randomization	Randomization was not relevant for the present study, because all the experiments in the present study were cell-based. Cells used in this study had to be differently treated and analyzed in parallel to minimize experimental variation.
Blinding	All the experiments were unblinded because these experiments were not susceptible to bias.

Reporting for specific materials, systems and methods

We require information from authors about some types of materials, experimental systems and methods used in many studies. Here, indicate whether each material, system or method listed is relevant to your study. If you are not sure if a list item applies to your research, read the appropriate section before selecting a response.

Materials & experimental systems

n/a	Involved in the study	
	x	Antibodies
	x	Eukaryotic cell lines
x		Palaeontology and archaeology
	x	Animals and other organisms
x		Clinical data
x		Dual use research of concern
x		Plants

Methods

n/a	Involved in the study	
x		ChIP-seq
x		Flow cytometry
x		MRI-based neuroimaging

Antibodies

Antibodies used

Antibodies used in this study were as follows: rabbit anti-STING (19851-1-AP, dilution 1:1000), rabbit anti-calnexin (10427-2-AP, dilution 1:1000) (Proteintech); rabbit anti-phospho-STING (D8F4W, dilution 1:1000 for western blot), rabbit anti-phospho-TBK1 (D52C2, dilution 1:1000), rabbit anti-IRF3 (D83B9, dilution 1:1000), and rabbit anti-phospho-IRF3 (4D4G, dilution 1:1000) (Cell Signaling Technology); rabbit anti-TBK1 (ab40676, dilution 1:1000) (Abcam); mouse anti-α-tubulin (10G10, dilution 1:1000) and mouse anti-FLAG (1E6, dilution 1:1000) (Wako); mouse anti-CH25H (J2617, dilution 1:100; Santa Cruz); Goat anti-Rabbit IgG (H + L) Mouse/Human ads-HRP (4050-05, dilution 1:10,000) and Goat anti-Mouse IgG (H + L) Human ads-HRP (1031-05, dilution 1:10,000) (SouthernBiotech); sheep anti-TGN38 (AHP499G, dilution 1:200) (Bio-Rad); mouse anti-GM130 (610823, dilution 1:4000) (BD Biosciences); Alexa 568-, 594-, or 647-conjugated secondary antibodies (A10037, A11016, A21448, dilution 1:1000) (Thermo Fisher Scientific).

Validation

All antibodies were validated by the vendors and documented with corresponding data sheets as follows.
rabbit anti-STING (19851-1-AP): validated for mouse STING by WB with cell lysate.
rabbit anti-calnexin (10427-2-AP): validated for mouse calnexin by WB with cell lysate.
anti-phospho-STING (D8F4W): validated for mouse pSTING by WB with cell lysate.
rabbit anti-phospho-TBK1 (D52C2): validated for mouse p TBK1 by WB with cell lysate.
rabbit anti-IRF3 (D83B9): validated for mouse IRF3 WB with cell lysate.
rabbit anti-phospho-IRF3 (4D4G): validated for mouse pIRF3 by WB with cell lysate.
anti-FLAG (1E6): validated by WB with lysate of the cells expressing Flag-tagged mouse STING.
mouse anti-CH25H (J2617): validated for mouse CH25H by WB with cell lysate.
sheep anti-TGN38 (AHP499G): validated for mouse TGN38 by IF.
mouse anti-GM130 (610823): validated for mouse TGN38 by IF.
Goat Anti-Rabbit IgG (H+L) Mouse/Human ads-HRP (Southern Biotech, 4050-05): validated for mouse Goat Anti-Rabbit IgG (H+L) Mouse/Human ads-HRP by WB with cell lysate.
Goat Anti-Mouse IgG (H+L) Human ads-HRP (Southern Biotech, 1031-05): validated for mouse Goat Anti-Mouse IgG (H+L) Human ads-HRP by WB with cell lysate.
donkey Alexa 488-, 594-, or 647- conjugated secondary antibodies (Thermo Fisher Scientific, A21202, A21203, A21206, A21207, A31573, A11016, A21448): validated for mouse Alexa 488-, 594-, or 647- conjugated secondary antibodies by IF with fixed cells.

Eukaryotic cell lines

Policy information about cell lines and Sex and Gender in Research

Cell line source(s)

Immortalized MEFs (described in (PMID: 36918692)). Human prostate cancer cells (PC3; ATCC, CRL-1435)

Authentication

N.A.

Mycoplasma contamination

Confirm that all cell lines were tested negative for mycoplasma contaminations.

Commonly misidentified lines
(See ICLAC register)

N.A.

Animals and other research organisms

Policy information about studies involving animals; ARRIVE guidelines recommended for reporting animal research, and Sex and Gender in Research

Laboratory animals

N.A.

Wild animals

N.A.

Reporting on sex

N.A.

Field-collected samples

N.A.

Ethics oversight	N.A.

Note that full information on the approval of the study protocol must also be provided in the manuscript.

Plants

Seed stocks	N.A.
Novel plant genotypes	N.A.
Authentication	N.A.
